# Asc-Seurat: analytical single-cell Seurat-based web application

**DOI:** 10.1186/s12859-021-04472-2

**Published:** 2021-11-18

**Authors:** W. J. Pereira, F. M. Almeida, D. Conde, K. M. Balmant, P. M. Triozzi, H. W. Schmidt, C. Dervinis, G. J. Pappas, M. Kirst

**Affiliations:** 1 School of Forest, Fisheries, and Geomatics Sciences, University of Florida, Gainesville, FL 32611 USA; 2 Department of Cell Biology, Institute of Biological Sciences, University of Brasília, Brasília, DF 70910-900 Brazil; 3 Genetics Institute, University of Florida, Gainesville, FL 32611 USA

**Keywords:** Single-cell RNA sequencing, scRNA-seq, Web application, Gene expression

## Abstract

**Background:**

Single-cell RNA sequencing (scRNA-seq) has revolutionized the study of transcriptomes, arising as a powerful tool for discovering and characterizing cell types and their developmental trajectories. However, scRNA-seq analysis is complex, requiring a continuous, iterative process to refine the data and uncover relevant biological information. A diversity of tools has been developed to address the multiple aspects of scRNA-seq data analysis. However, an easy-to-use web application capable of conducting all critical steps of scRNA-seq data analysis is still lacking.

**Summary:**

We present Asc-Seurat, a feature-rich workbench, providing an user-friendly and easy-to-install web application encapsulating tools for an all-encompassing and fluid scRNA-seq data analysis. Asc-Seurat implements functions from the Seurat package for quality control, clustering, and genes differential expression. In addition, Asc-Seurat provides a pseudotime module containing dozens of models for the trajectory inference and a functional annotation module that allows recovering gene annotation and detecting gene ontology enriched terms. We showcase Asc-Seurat’s capabilities by analyzing a peripheral blood mononuclear cell dataset.

**Conclusions:**

Asc-Seurat is a comprehensive workbench providing an accessible graphical interface for scRNA-seq analysis by biologists. Asc-Seurat significantly reduces the time and effort required to analyze and interpret the information in scRNA-seq datasets.

**Supplementary Information:**

The online version contains supplementary material available at 10.1186/s12859-021-04472-2.

## Background

RNA sequencing (RNA-seq) was developed more than a decade ago and has since revolutionized our understanding of biology [1]. During this time, RNA-seq from bulk tissue has become the standard approach to investigate the transcriptome of a wide range of organisms. However, bulk tissue RNA-seq is not suitable for the characterization of rare cell types or distinguishing cell to cell variability [2]. Due to these limitations and recent technological advances, a growing number of studies have adopted single-cell RNA sequencing (scRNA-seq) as a replacement for bulk tissue experiments [1].

Single-cell technologies dramatically enhanced our capacity to characterize tissues and their cell types. By quantifying individual cells' gene expression, scRNA-seq substantially increases the resolution of transcriptome profiles by disentangling the data derived from each cell [1–3]. The application of scRNA-seq to humans, animals, and plants has led to the discovery of new cell types and a better understanding of organismal development. Moreover, single-cell analysis is crucial in uncovering cellular heterogeneity, identifying rare cell populations, distinct cell-lineage trajectories, and mechanisms involved in complex cellular processes [2]. Single-cell RNA-seq public resources are expanding rapidly, and the accumulation of massive cellular transcriptome data is in progress [2]. The EMBL-EBI Single Cell Expression Atlas [4] currently contains more than 180 studies from 14 species (February 2021).

Due to the great potential of scRNA-seq technology, several computational tools have been developed to address the different aspects of data analysis. While these tools apply various programming languages, the most widely used are written in R or Python [4]. Among them, Seurat [5] is an R package widely used for scRNA-seq data processing, cell clustering, and detecting differentially expressed genes (DEGs) from single or multiple samples. Follow-up analyses, including single-cell lineage development trajectory inference (TI) tools, are provided by other software such as the dynverse collection of R packages [6]. Using dynverse, multiple TI models can be evaluated, and DEGs within the inferred trajectory can be identified.

Seurat's and dynverse's functions aim to be simple to use, and their results are reported in a series of plots and tables that are intuitive to interpret. Nevertheless, the dependency on command-line interface and R programming language proficiency poses a significant barrier for researchers with limited computational expertise, impacting their capacity to explore the data. Besides, each analytical step outcome is strongly influenced by data quality and execution parameters, requiring the continuous manipulation of these parameters and reevaluation of subsets of data to uncover their biological meaning. Tools that simplify the iterative scRNA-seq analysis process and the integration across analysis platforms are essential for biologists. Recently, several such tools have been developed for scRNA-seq [7–14]. While these tools aim to solve similar issues—providing a graphical user interface as an alternative to command-line—they often address different aspects of the analysis, contain different underlying software, and may not encompass all analytical steps. For example, SCiAp [13] contains many of the most common software used in scRNA-seq. Still, its reliance on the Galaxy framework [15] makes its installation and use difficult for scientists without a computational background. ASAP [7], BingleSeq [10], and Seurat Wizard (now part of NASQAR [11]) offer Seurat’s capabilities, but lack TI analysis support. Others, such as SC1 [14] and PIVOT [8], provide tools for many of the analytical steps but are based on methods that are less widely adopted by the community than the Seurat toolkit and only offer one model for TI. Tools such as alona [12] do not provide the interactive selection of parameters and evaluation of results. Therefore, a complete workflow encompassing the most critical stages of data analysis and their interpretation is still lacking.

Here we present Asc-Seurat (Analytical single-cell Seurat-based web application), an easy-to-install interactive web application that provides a comprehensive scRNA-seq analysis workbench. Below, we showcase Asc-Seurat’s architecture and capabilities by analyzing two publicly available datasets.

## Implementation

### Overview

**Fig. 1:**
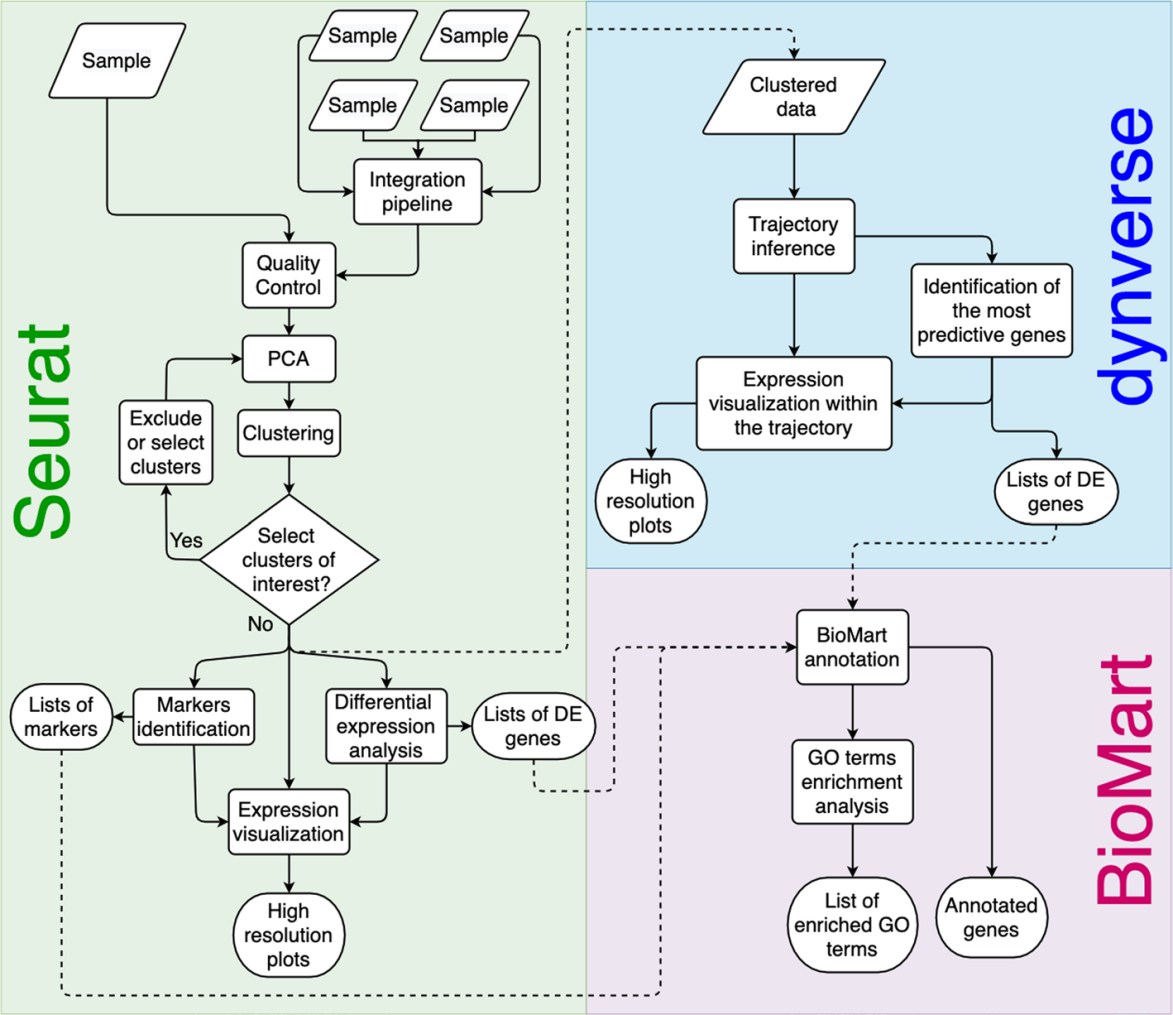
Asc-Seurat workflow overview. Asc-Seurat is built on three analytical cores. Using Seurat, users explore scRNA-seq data to identify cell types, markers, and DEGs. Dynverse allows the evaluation and visualization of developmental trajectories and identifies DEGs on these trajectories. Asc-Seurat also implements BioMart for functional annotation and GO term enrichment analysis

Asc-Seurat is a modular web application implemented using R language and user interface provided by the Shiny framework [16] and R [17]. The main modules are described in Fig. 1 and encapsulate several analytical procedures including: (1) the algorithmic capabilities of Seurat for cell clustering, differential expression analysis, and expression visualization; (2) Dynverse functionalities offering dozens of models for TI, combined with (3) gene functional annotation using BioMart [18] via the biomaRt package [19]. We provide a platform-independent installation that can handle all dependencies and configuration for software execution using the Docker virtualization platform.

After the installation, users can access and use Asc-Seurat via their web browser. For a complete description of the installation steps, including the command line to download and execute the Asc-Seurat’s Docker image, visit Asc-Seurat’s documentation [20].

### Input

During the first execution of Asc-Seurat in a working directory, two folders are created: (1) *data/*, which is used as the location for the input files, and (2) *RDS_files****/*** that is used to save intermediate files, allowing their usage through the multiple modules of the application.

Asc-Seurat requires, as input files, the feature-barcode matrices generated by Cell Ranger (10× Genomics [21]). If using alternative software, users can convert the output to the Cell Ranger format by applying other freely available tools. For example, the DropletUtils package [22, 23] can be used to generate the 10× Genomics feature-barcode matrices from any count matrix.

### Clustering module (Seurat): quality control, clustering, differential expression analysis, and visualization tools

**Fig. 2:**
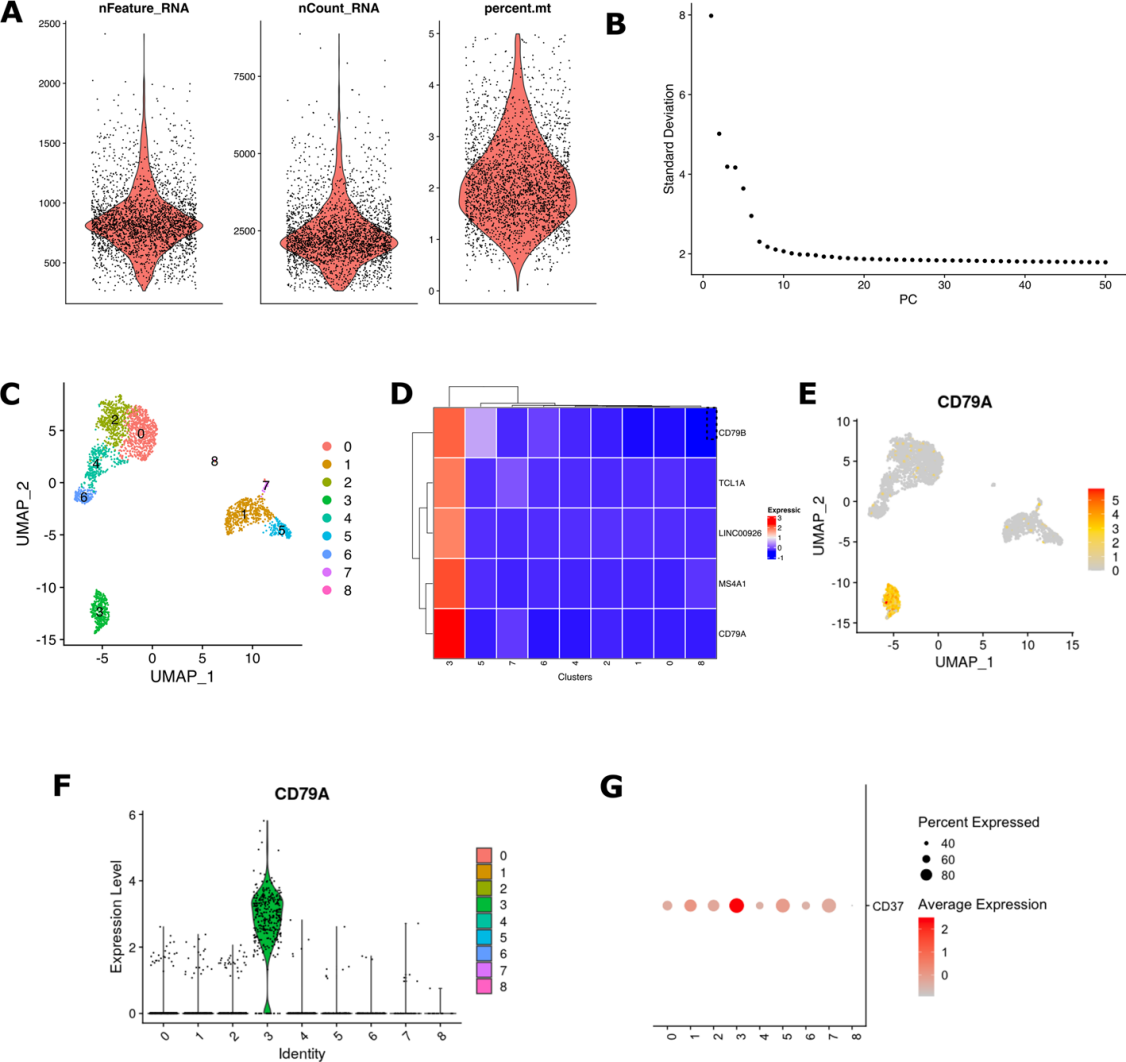
Asc-Seurat iteratively generates supporting plots based on the Seurat package through the analysis steps. Several plots are generated in each step of the analysis, allowing quick interpretation and parameter tuning. **A** Violin plot showing the distribution of the cells on the loaded data, allowing the exclusion of low-quality cells. **B** Elbow plot generated to support the choice of the number of PCs to be used during clustering. **C** UMAP plot showing the distribution of cells and clusters. **D** Heatmap showing the averaged gene expression in each cluster, providing a quick overview and selection of genes for further visual exploration. **E** UMAP plot showing the expression profile of a gene at the cell level. **F**, **G** Violin and dot plot, respectively, showing the expression profile at the cluster level and facilitating the inter-cluster comparison

The Asc-Seurat’s clustering module is based on the Seurat (v4) package and provides a rich graphical user interface (GUI), allowing the interactive manipulation of the data and execution of many of Seurat’s functions. Asc-Seurat can be used to analyze an individual sample or analyze multiple samples by deploying Seurat’s integration algorithm [5]. Through its GUI, Asc-Seurat provides all steps for: (1) quality control, by the exclusion of low-quality cells and potential doublets; (2) data normalization, including log normalization and the SCTransform [24], (3) dimension reduction via principal component analysis (PCA), (4) clustering of the cell populations, including the selection or exclusion of clusters and re-clustering, and (5) differential expression analysis to identify markers for specific clusters, or DEGs among clusters or samples in a cluster when using the sample integration method (Fig. 2).

During these analytical steps, several plots are generated in real-time, allowing users to inspect the results and, if necessary, to adjust the parameters and re-execute the analysis accordingly. Moreover, users can download all results as comma-separated values (CSV), high-resolution plots, or an intermediary file containing the clustered data to use as input in the other modules of Asc-Seurat.

Once clusters are defined, users can plot the gene expression at the cluster or cell level. To visualize the expression plots, users provide a list of gene identifiers. Usually, these genes are known markers for specific cell types or DEGs identified using Asc-Seurat. A heatmap is generated showing the average of the expression of each gene per cluster, with the possibility to select groups of genes for further exploration. For each selected gene, a uniform manifold approximation and projection (UMAP) plot showing its expression in each cell is generated, as well as a violin plot and a dot plot showing the expression profile in each cluster (Fig. 2).

**Fig. 3:**
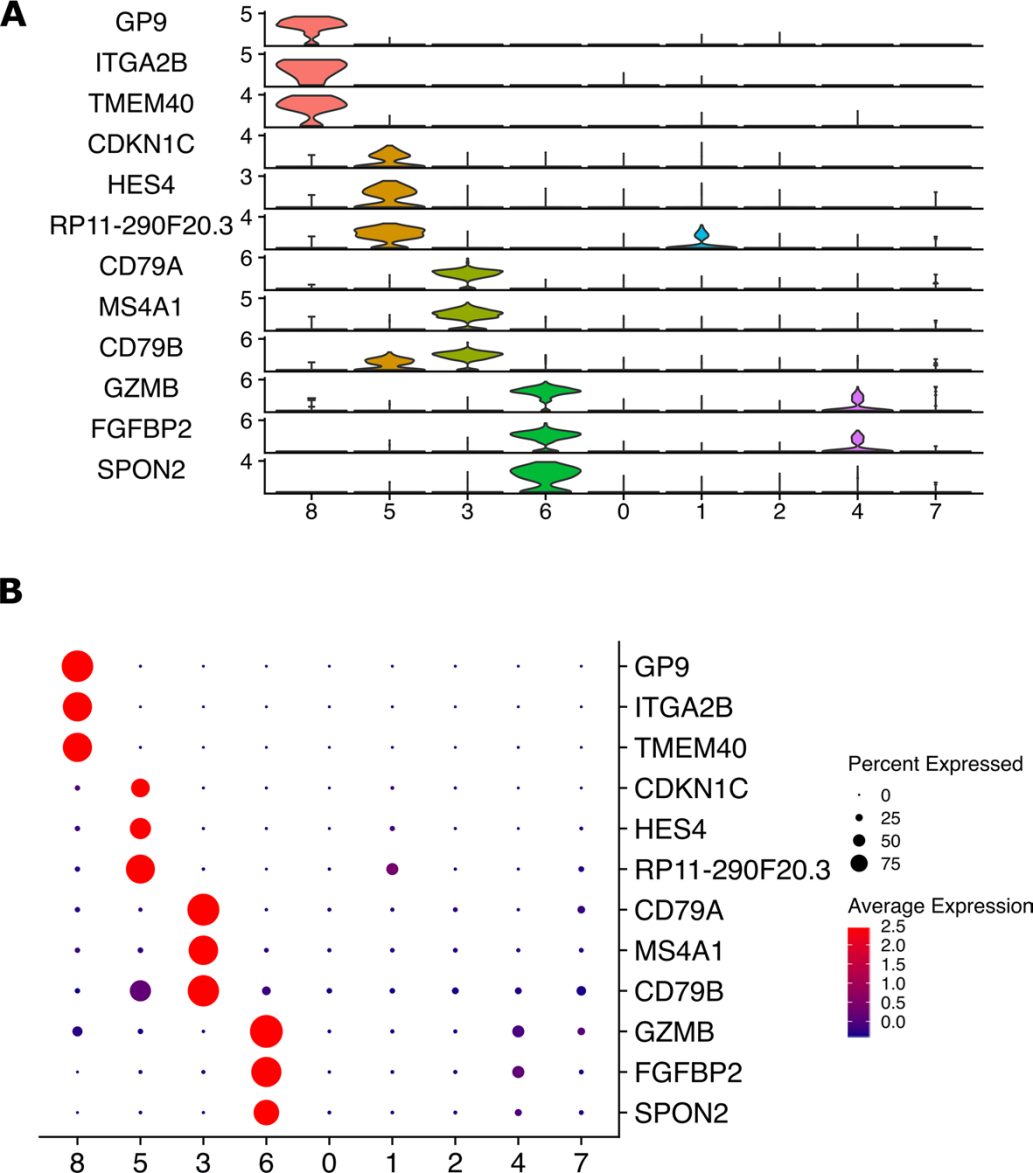
Asc-Seurat enables the comparison of the expression profile of multiple genes by generating stacked violin plots (**A**) and multiple-genes dot plots (**B**). Both **A** and **B** show the expression profiles of three gene markers for each of the clusters 8, 5, 3, and 6

In addition to the plots described above, Asc-Seurat also includes the option to generate “stacked-violin” plots to compare the expression distribution of multiple genes in distinct clusters (Fig. 3A). Moreover, dot plots comparing multiple genes can also be generated (Fig. 3B). To generate these plots, users only need to input the clustered dataset (as generated by Asc-Seurat) and select the order in which genes and clusters should be displayed in the y-axis and x-axis, respectively (Fig. 3).

### Trajectory inference module (dynverse): trajectory inference, visualization, and identification of genes governing the trajectory

Besides the analytical capabilities of the Seurat package, Asc-Seurat also allows the inference of developmental trajectories by taking advantage of dozens of TI models implemented in the dynverse collection of R packages [6]. Users are encouraged to review Saelens et al., 2019 [6] to compare the TI models. For this analysis, users provide the clustered dataset and select the model to be executed. Users can also provide the clusters expected to be at the beginning and end of the trajectory, according to the lineage expectations for the cell population. Note that this information is optional for some models but required for others.

**Fig. 4:**
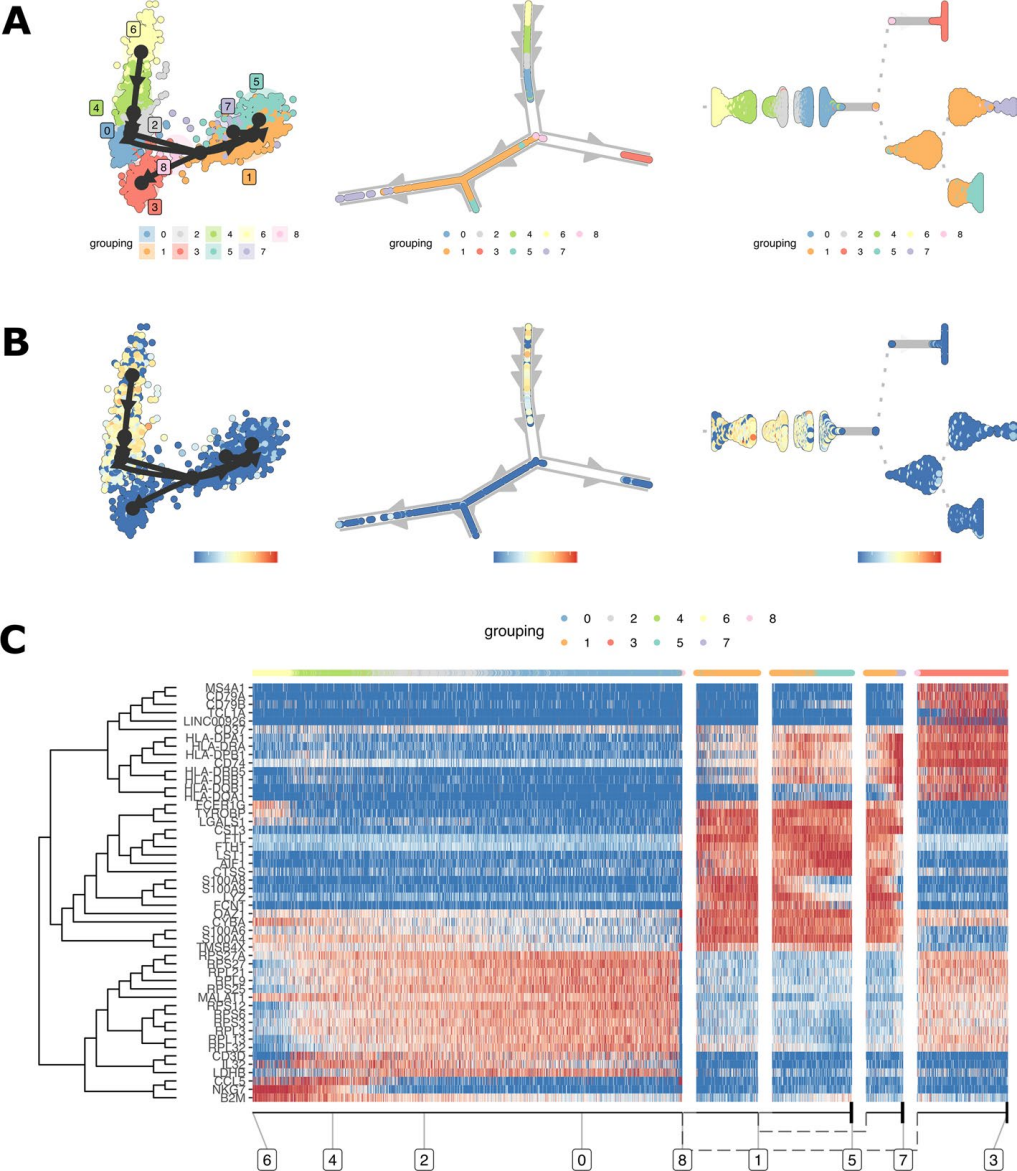
Asc-Seurat provides options for visualization of gene expression profiles within the inferred trajectories. For each trajectory, three visualization plots are generated, facilitating its interpretation (**A**). Users can also visualize the expression profile of any gene of interest within the trajectories (**B**) or as a heatmap (**C**). In **A** and **B**, arrows indicate the direction of the trajectory. In **C**, at the bottom of the heatmap, the dashed lines connect the clusters (shown inside squares) according to different branches of the inferred trajectory

Users also need to visit the documentation of their model of choice to understand the required inputs and estimate the necessary computational resources and amount of time to execute the model in their datasets. Three trajectory representations are generated after executing the selected model, showing the distribution of cells in the inferred trajectory according to the clustering (Fig. 4A). Alternatively, the cells can be colored by sample if an integrated dataset is used. Users can also select genes to create plots showing their expression in the cells within the trajectory (Fig. 4B).

Inferred trajectories can be further analyzed to identify genes that are critical in defining their topology. Genes involved in regulating the transition among cell types or subtypes within a trajectory are often of interest. Asc-Seurat allows users to search for such genes by deploying the dynfeature package [6] to classify the genes accordingly with their importance in defining the inferred trajectory. In addition, users can visualize the expression of selected genes within the trajectory as a heatmap showing the expression per cell following their order in the trajectory (Fig. 4C).

### Gene annotation module: gathering gene annotation information and gene ontology (GO) enrichment analysis

After identifying genes of interest using the clustering and trajectory inference modules, users can obtain information about their function to interpret the data further and generate follow-up hypotheses. Asc-Seurat provides this capability to hundreds of species by deploying BioMart [18]. Using a list of genes as input, it is possible to recover their functional annotation from multiple databases, including Gene Ontology (GO) terms [25]. The table containing the genes annotation can then be explored within the application or downloaded.

Users can also perform GO term enrichment analysis to identify over (or under) representation in a set of genes of interest compared to the expected frequency based on the annotation of a reference group of genes. For example, to investigate the cell type represented by a cluster, users can perform GO enrichment analysis to identify terms over-represented in the set of markers identified using Asc-Seurat for that cluster. This analysis is particularly interesting when a set of validated gene markers is not available in the literature to assign the cluster’s cell-type, and the information needs to be gathered from the dataset at hand. The list of enriched GO terms can be downloaded, and a plot showing the most enriched terms in each category can be download as a high-resolution image.

For a step-by-step utilization guide refer to Asc-Seurat’s webpage [20].

## Results

To demonstrate Asc-Seurat's functionalities, we analyzed the publicly available 10× Genomics’ 3k Peripheral Blood Mononuclear Cells (PBMC) dataset [26], showcasing the analysis of an individual sample. In addition, we used a second PBMC dataset to demonstrate the analysis integrating multiple samples in Asc-Seurat. The second PBMC dataset was generated by Hang et al., 2018 [27] and distributed as part of the SeuratData package [28]. It contains two samples and approximately fourteen thousand cells. Both samples contain a pool of PBMC cells from eight patients. However, one sample was stimulated by treatment with IFN-β (Treatment) while the second sample is a control (Control). Moreover, we provide a detailed comparison among the available web applications. While these web applications partly overlap with Asc-Seurat’s capabilities, none to date comprises the range of essential tools available in Asc-Seurat.

### Asc-Seurat use case 1—analysis of an individual sample

#### Loading the data, quality control, data normalization and clustering

For analysis using Asc-Seurat, all scRNA-seq datasets should be stored in a subdirectory inside the directory *data/*, generated during the installation. Asc-Seurat’s interface will display compatible files stored within the *data/* folder, from where the data of interest can be selected. Next, users can provide a name for the project and define the initial parameters to select cells to be loaded in the web application. For the 10× Genomics’ PBMC dataset, we selected only cells expressing at least 200 genes, and only genes expressed in three or more cells. These parameters are fully adjusteable in Asc-Seurat.

After loading the dataset, a violin plot shows the distribution of the number of expressed genes, the number of Unique Molecular Identifiers or independent transcript, and the percentage of mitochondrial genes detected in each cell (Additional file 1: Fig. S1). Users can then define more restrictive parameters to remove undesirable cells based on the observed distribution. For the PBMC dataset, we selected only cells expressing more than 250 and less than 2500 genes. We also excluded cells with more than 5% of transcripts from mitochondrial origin (Additional file 1: Fig. S1).

Subsequently, users select the normalization procedure to be applied to the dataset (log-normalization or SCTransform), as well as parameters for the dimension reduction using PCA. For the PBMC dataset, we performed the log normalization using a scale factor of 10000. Also, the dimension reduction by PCA was performed using the 2000 most variable genes selected by the “vst” method. Default values sufficient for most of the datasets are provided. After executing the PCA, an elbow plot is generated to help users define how many principal components (PCs) should be used for clustering the data. For the PBMC dataset, we used the first 10 PCs (Additional file 1: Fig. S2).

Before executing the clustering step, it is necessary to inform the resolution parameter, which strongly influences the profile and number of clusters identified for a dataset. Selecting larger values will favor splitting cells into more clusters while selecting smaller ones has the opposite effect. For the PBMC dataset, a resolution of 0.5 was selected, and nine clusters were identified (Additional file 1: Fig. S3).

#### Differential expression analysis and gene marker identification

Asc-Seurat provides an assortment of algorithms to identify gene markers for individual clusters or DEGs among clusters. As an example, we searched for gene markers for cluster 3 of the PBMC dataset. When using the non-parametric Wilcoxon rank-sum test, filtering for genes expressed in at least 10% of the cells in the cluster, with a (log) fold change higher than 0.25 and an adjusted *p* value smaller than 0.05, 397 gene markers were identified (Additional file 1: Fig. S4).

#### Gene expression visualization

Asc-Seurat provides a variety of plots for gene expression visualization. From a list of selected genes, it is possible to visualize in a heatmap the averaged expression of each gene in each cluster (Fig. 2D) and, in a UMAP plot, the expression of the gene at the cell level (Fig. 2E). Moreover, violin plots (Fig. 2F) and dot plots (Fig. 2G) provide a tool for the visualization of the expression profile of each cluster, with emphasis on the inter-cluster comparison. As an example, we generated a heatmap plot for the five most significant markers identified in cluster 3 (Additional file 1: Fig. S5) and show their expression profile at the cell level (Additional file 1: Fig. S6) and the cluster level (Additional file 1: Fig. S7).

#### Trajectory inference and identification of genes defining the trajectory

Identifying genes affecting the developmental trajectory is critical for understanding how cells differentiate from one type to another. Therefore, after exploring the clusters, users may want to identify the developmental trajectory between cells in different clusters, subclusters, or states (i.e., cells responding to treatment). Moreover, it can be of interest to identify genes that vary in their expression within a trajectory.

To infer a developmental trajectory, users can either execute the capabilities of the embedded slingshot R package or select from dozens of models contained in dynverse. The choice of the model is important since some models are designed to perform well when the inferred trajectory follows a specific topology but perform poorly in others [6]. After executing the analysis, three plots showing different inferred trajectory representations are generated (Fig. 4A). For the PBMC dataset, a developmental trajectory containing three lineages was identified using the nine clusters as input (Additional file 1: Fig. S8).

After inferring the developmental trajectory, it is possible to visualize the expression of genes of interest in the cells within the trajectory. Asc-Seurat provides two options for the visualization of gene expression within the trajectory: (1) the visualization of the same three trajectories represented in Fig. 4A, but colored by the gene expression (Fig. 4B), and (2) a heatmap displaying the expression of genes in each cell, ordered by the cell position within the trajectory (Fig. 4C).

For the PBMC dataset, we opted to show the 50 most significant DEGs within the trajectory, as ranked by their importance value estimated by dynverse (Additional file 1: Fig. S9). We selected three representative genes to show their expression using the three approaches mentioned above; *NKG7*, expressed in cells at the beginning of the trajectory; and *LST1* and *MS4A1*, expressed in alternative branches in later parts of the trajectory (Additional file 1: Fig. S10).

#### Recovering functional annotation information and GO enrichment analysis

In many instances, users are interested in obtaining more information about a gene, or a set of genes, to support the interpretation of the data and the development of new hypotheses. For example, Asc-Seurat produces lists of gene markers, DEGs, and DEGs within a trajectory that might be of particular interest. By providing the capacity of querying BioMart servers via the biomaRt package [19], Asc-Seurat allows recovering the functional annotation for genes of several species. Furthermore, GO term enrichment analysis is also provided to verify if one or more GO terms are over-represented or under-represented in a set of selected genes.

As an example, we executed the GO term enrichment analysis for the set of 50 most important DEGs within the trajectory inferred for the PBMC dataset, according to dynfeature’s importance value, using all expressed genes as the universe (background) of the analysis. We identified two terms related to the immune system as significantly enriched (Additional file 1: Fig. S11).

### Asc-Seurat use case 2—analysis of multiple samples using the integration approach

Using Seurat’s integration approach, the analysis of multiple samples is, in many ways, similar to the analysis of an individual sample. Therefore, while mentioning all required steps, we will focus on the steps where the analysis of multiple samples diverges the most when using Asc-Seurat.

#### Data loading, quality control, normalization, and integration

For the integration of multiple samples, the steps of loading the data are different from when using a single sample. Users still need to add their datasets in the *data/* directory, creating a subdirectory for each sample. However, users also need to provide a configuration file containing the parameter values for each sample. An example of the configuration file is generated during the installation. We also provide the configuration file used to integrate the two samples from the PBMC IFN-β dataset in Additional file 1: Table S1. These parameters include the name of the sample and the values used in the quality control. Therefore, users need to explore each sample individually and define these parameters before starting the integration of the samples. Moreover, within Asc-Seurat’s interface, users also need to select the normalization to be performed in the dataset and other parameters for the integration. The selected parameters for the PBMC IFN-β dataset are shown in Additional file 1: Fig. S12 and are extensively described in Asc-Seurat’s documentation.

#### Clustering, differential expression, and expression visualization

After the integration is completed, the analysis is similar to the described above for a single sample. A violin plot showing the distribution of cells is generated, and users can select more strict filtering parameters, then perform the PCA and clustering. For the PBMC IFN-β, we did not apply cell filtering after the integration. Next, 20 PCs and a resolution of 0.5 were used for clustering, and 15 clusters were identified (Additional file 1: Fig. S13 and Additional file 1: Fig. S14).

Two significant differences exist when searching for gene markers or DEGs using multiple samples. First, the search for gene markers identifies those that are also conserved among samples. Second, it is possible to identify DEGs between samples for each cluster. For example, we identified 182 DEGs between the treatment and control for cluster 7 (Additional file 1: Fig. S15).

In terms of expression visualization, the main difference of using an integrated dataset is that the UMAP plot showing the gene expression per cell is separated by sample, allowing a visual comparison between them. For example, we selected the five most DEGs that are more highly expressed in the treatment sample for cluster 7 (Additional file 1: Fig. S16 and Additional file 1: Fig. S17).

#### Trajectory inference and identification of genes defining the trajectory

For the trajectory inference, the analysis is conducted similarly for both an individual sample or an integrated dataset containing multiple samples. The only difference is that the user can indicate that the dataset contains multiple samples and, therefore, visualize the distribution of the cells within the trajectory colored by sample. The distribution of the cells of the PCMB IFN-β within the trajectory and colored by sample is shown in Additional file 1: Fig. S18.

## Conclusions

With the increasing usage of scRNA-seq to investigate the transcriptome, there is a critical need to generate tools that allow biologists to efficiently perform data analysis and interpretation. Here we described Asc-Seurat, a complete workbench for scRNA-seq with a rich and easy-to-use interface that can be used by all biologists, regardless of their computational expertise. In Table 1, we describe a comparison of Asc-Seurat's capabilities relative to the most relevant web applications currently available.

**Table 1:** Comparison of Asc-Seurat's capabilities with the most relevant web applications currently available

	Asc-Seurat	NASQR [11]	SCiAp [13]	PIVOT [8]	SC1 [14]	Stream [9]	alona [12]	ASAP [7]	BingleSeq [10]
*Usability*									
Is it a web application?	Yes	Yes	Yes (2)	Yes	Yes	Yes	Yes	Yes	Yes
Easy to install (Docker)?	Yes	Yes	No	Yes	No	Yes	No	No	No
Easy to use by wet-lab biologists?	Yes	Yes	Yes (3)	Yes	Yes	Yes	Yes	Yes	Yes
*Clustering*									
Does the clustering?	Yes	Yes	Yes	Yes	Yes	No	Yes	Yes	Yes
Does the clustering using Seurat?	Yes	Yes	Yes	No	No	No	Yes	Yes	Yes
Is it capable of integrating multiple samples?	Yes	Yes (1)	NA	Yes	Yes (7)	No	No	NA	No
Offers the SCtransform normalization?	Yes	Yes	No	No	No	No	No	No	No
Allows the filtering of clusters of interest?	Yes	No	Yes (4)	No	NA	No	No	No	No
High-quality plots of the clustering and expression?	Yes	Yes	Yes	Yes (6)	Yes	No	Yes	Yes	Yes
*Trajectory inference*									
Performs trajectory inference (TI)?	Yes	No	Yes	Yes	Yes	Yes	No	No	No
Offers multiple models for TI?	Yes	No	Yes	No	No	No	No	No	No
Differential Expression analysis within the trajectory?	Yes	No	Yes	Yes	No	Yes	No	No	No
Expression visualization within the trajectory?	Yes	No	Yes	Yes	No	Yes	No	No	No
*Annotation*									
Gene annotation?	Yes	Yes	Yes (5)	Yes	Yes	No	No	Yes	Yes (8)
GO terms enrichment	Yes	Yes	Yes (5)	Yes	Yes	No	No	Yes	No

NA (not available): the authors could not address this question with the information available in the manual, tutorials, or publications related to the web application. 1—It is not clear how the integration is performed, but it appears not to use Seurat's integration approach. 2—It is based on the Galaxy framework. 3—Requires training in the Galaxy framework. 4—Users need to provide the cell IDs manually for exclusion. 5—It is possible when using other Galaxy modules. 6—Does not provide UMAP, which has become the most used visualization method for scRNA-seq clustered data. 7—It is capable of analyzing multiple samples. However, it seems not to apply Seurat's integration approach. 8—Available only for a limited set of model organisms

## Availability and requirements

- Project name: Asc-Seurat (Analytical single-cell Seurat-based web application)
- Project home page: https://asc-seurat.readthedocs.io/en/latest/index.html; https://github.com/KirstLab/asc_seurat/
- Operating system(s): Platform independent
- Programming language: R (Shiny)
- Other requirements: Docker
- License: GNU GPL-3.0
- Any restrictions to use by non-academics: None.

## Supplementary Information

**Additional file 1: Asc-Seurat.** The Additional file 1 contains Table S1 and the Figures S1 to S18.

## Data Availability

The two datasets analyzed during the current study are publicly available. The first dataset, 3k Peripheral Blood Mononuclear Cells (PBMC), is distributed by 10× Genomics [26]. The second, also a PBMC dataset was generated by Hang et al., 2018 [27] and distributed as part of the SeuratData package [28].
