# Supplementary material for: Asc-Seurat: analytical single-cell Seurat-based web application
Source: BMC Bioinformatics. 2021 Nov 18;22:556. doi: 10.1186/s12859-021-04472-2 (PMC8600690; doi:10.1186/s12859-021-04472-2)
Supplement: Supplementary file 1 — Additional file 1: Asc-Seurat. The Additional file 1 contains Table S1 and the Figures S1 to S18. [file 12859_2021_4472_MOESM1_ESM.docx]

# Asc-Seurat – Supplementary Material

Pereira WJ^1*^, Almeida FM^2^, Balmant KM^1^, Conde D^1^, Triozzi PM^1^, Schmidt HW^1^, Dervinis C^1^, Pappas Jr GJ^2^, Kirst M^1,3^

^1^ School of Forest, Fisheries, and Geomatics Sciences, University of Florida, Gainesville, FL 32611, USA.

^2^ Department of Cell Biology, Institute of Biological Sciences, University of Brasília, Brasília, DF 70910-900, Brazil.

^3^ Genetics Institute, University of Florida, Gainesville, FL 32611, USA.

*** Correspondent author:** wendelljpereira@gmail.com

**Table S1. Example of a configuration file required for the integration of multiple samples using Asc-Seurat.**

| **Subdirectory name (must be inside data/)** | **Sample name (any name of your choice)** | **Minimum number of cells expressing a gene so the genes is included** | **Minimum number of genes a cell must express to be included** | **Maximum number of genes a cell can express and still be included** | **Maximum percentage of genes belonging to the mitochondrial genome** |
| --- | --- | --- | --- | --- | --- |
| example_PBMC_control | Control | 3 | 250 | 2500 | 5 |
| example_PBMC_treatment | Treatment | 3 | 250 | 2500 | 5 |

**
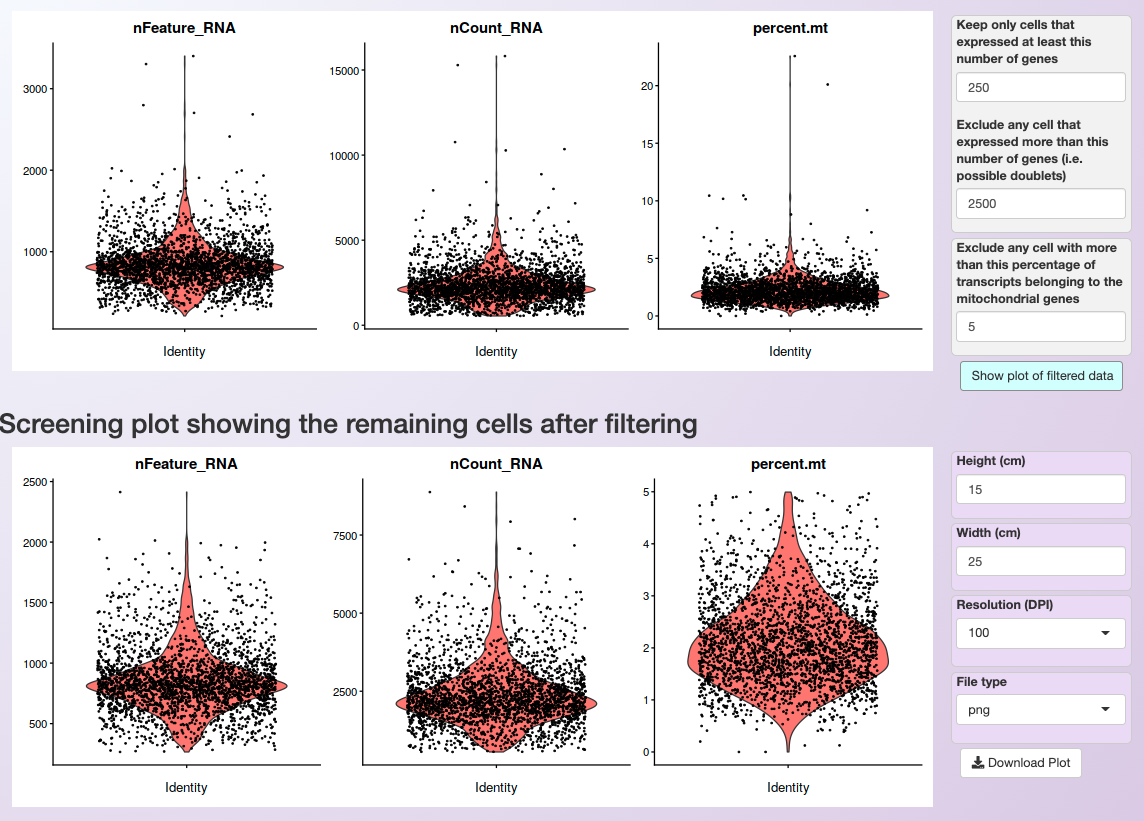
**

**Figure S1. Data filtering.** Violin plots show the PBMC cells' distribution before (upper panel) and after (lower panel) the filtering of undesirable cells.


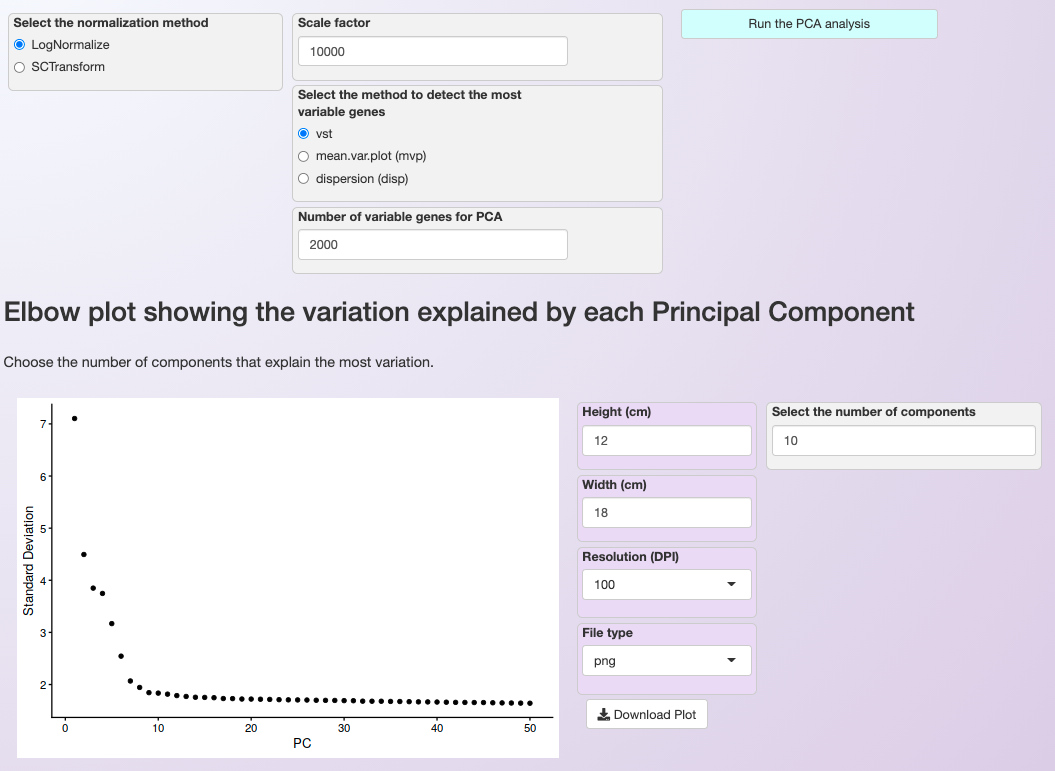


**Figure S2. Asc-Seurat integrates the normalization and PCA in one step.** After choosing the required parameters (top), users can trigger the analysis, and Asc-Seurat will execute the normalization and PCA. An elbow plot (bottom left) is then generated to help users select the number of PCs used in the clustering. For the PBMC dataset, the first 10 PCs (bottom right) were selected.


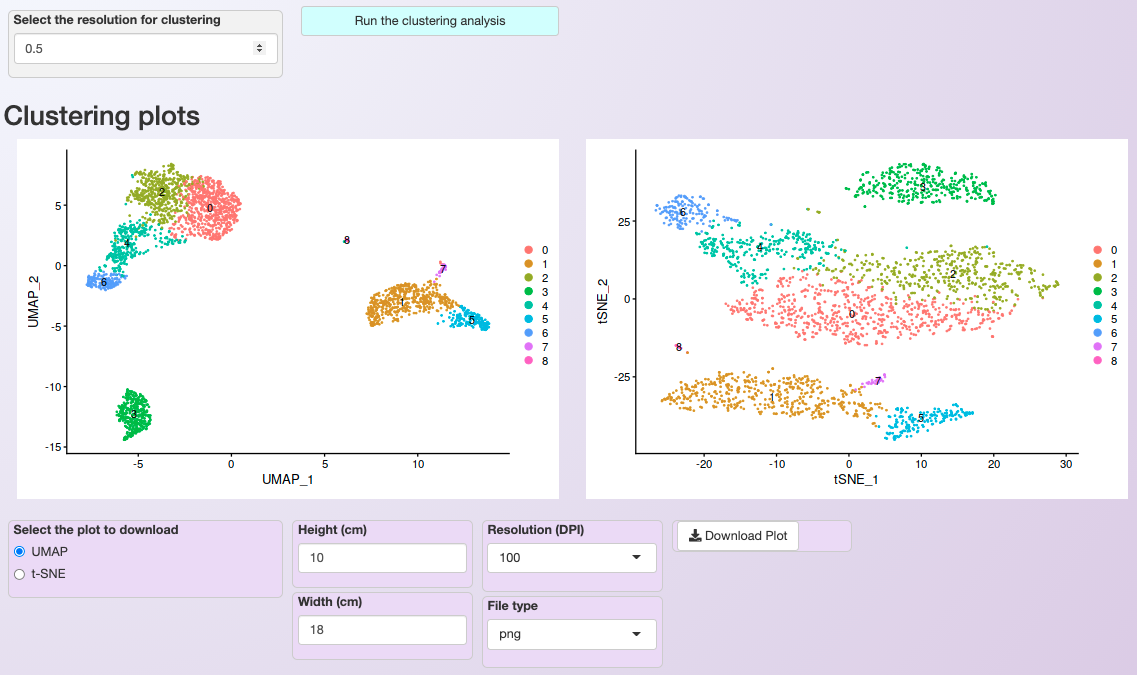


**Figure S3. Cluster visualization in Asc-Seurat.** Clustering is strongly affected by the resolution value. For the PBMC dataset, a resolution of 0.5 was defined (top). Two plots are generated for clustering visualization. The first plot is generated using the Uniform Manifold Approximation and Projection (UMAP) technique (bottom left). The second deploys the t-distributed Stochastic Neighbor Embedding (t-SNE) method (bottom right). Nine clusters were obtained for the PBMC dataset.


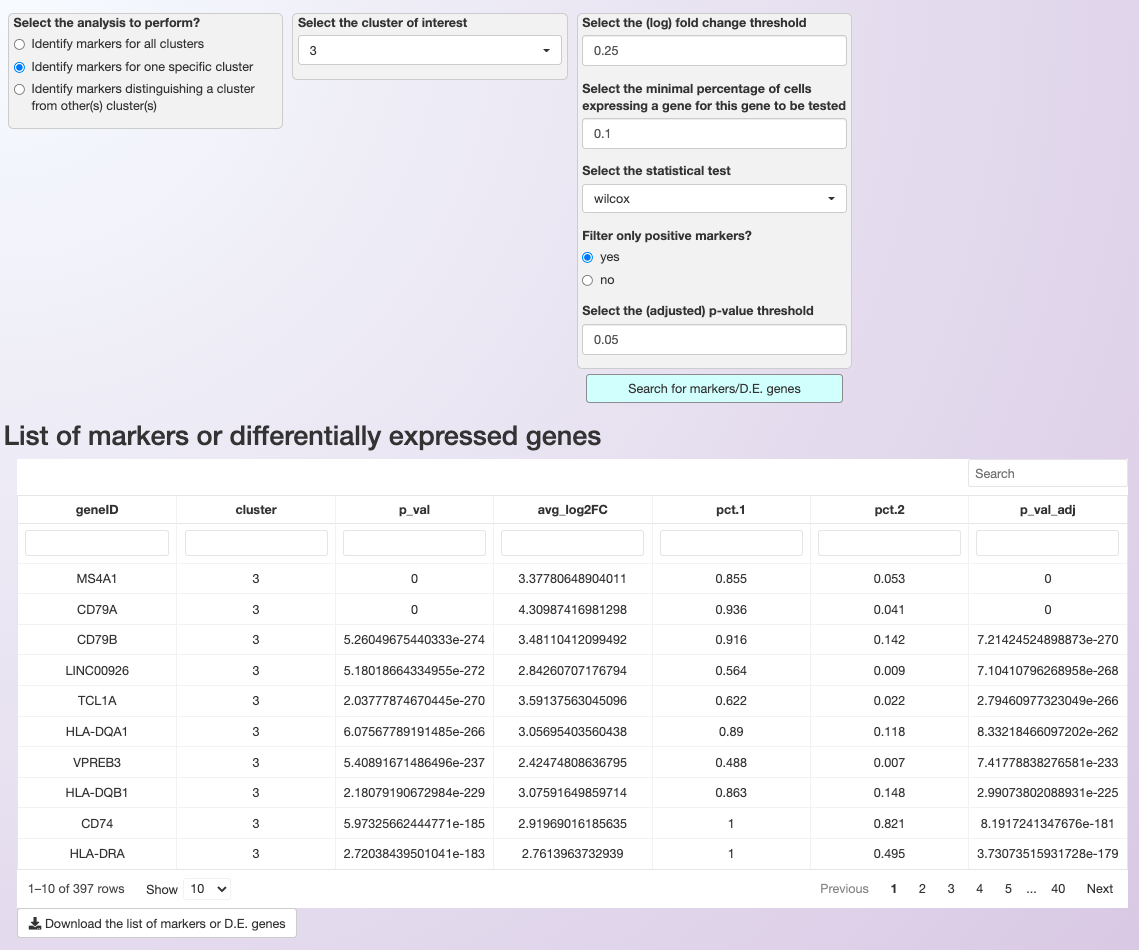


**Figure S4. Asc-Seurat allows the identification of gene markers and DEGs**. Users can search for gene markers for all clusters, for a specific cluster, or search for DEGs among specific clusters (top). An iterative table displaying the significant genes is generated (bottom), and users can download the list of markers, or DEGs, as a CSV (comma-separated values) file.

**
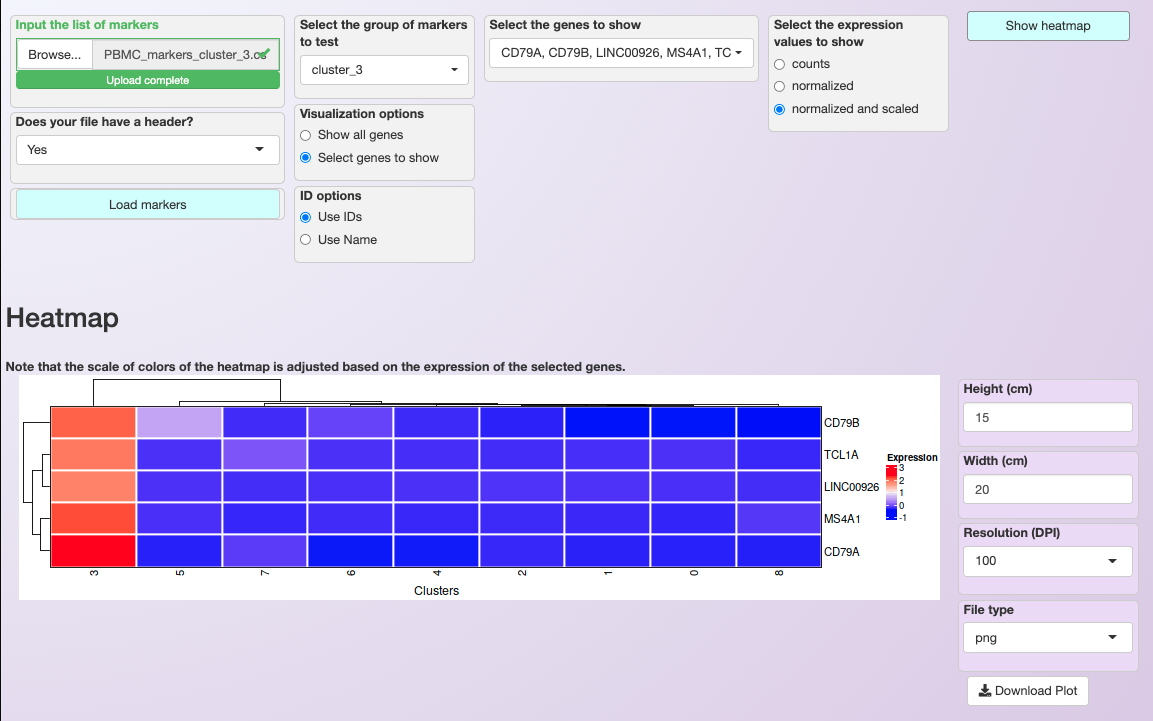
**

**Figure S5. Asc-Seurat provides a heatmap to allow the comparison of the gene expression profile among clusters.** Users can provide a list of genes as a CSV file, filter those by group, and/or select the genes of interest (top). The heatmap shows the average expression of the selected genes in each cluster. Moreover, Asc-Seurat groups genes with similar expression using a hierarchical clustering algorithm. Asc-Seurat adjusts the plot height based on the number of selected genes, and it can be readily downloaded (bottom).


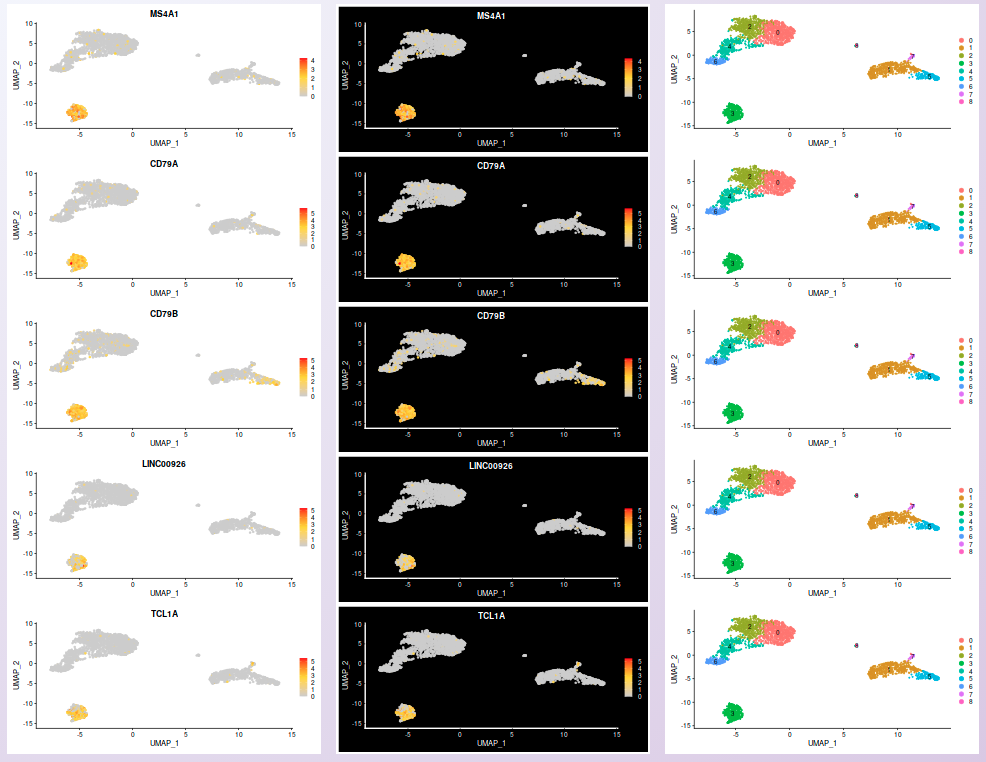


**Figure S6. Asc-Seurat allows the visualization of the gene expression at the cell level by deploying Seurat’s feature plots.** Using the same five markers selected for cluster 3 on the heatmap, it is possible to observe that the expression of these genes is, as expected, higher among cells in that, with a large percentage of cells of this cluster expressing the selected genes.


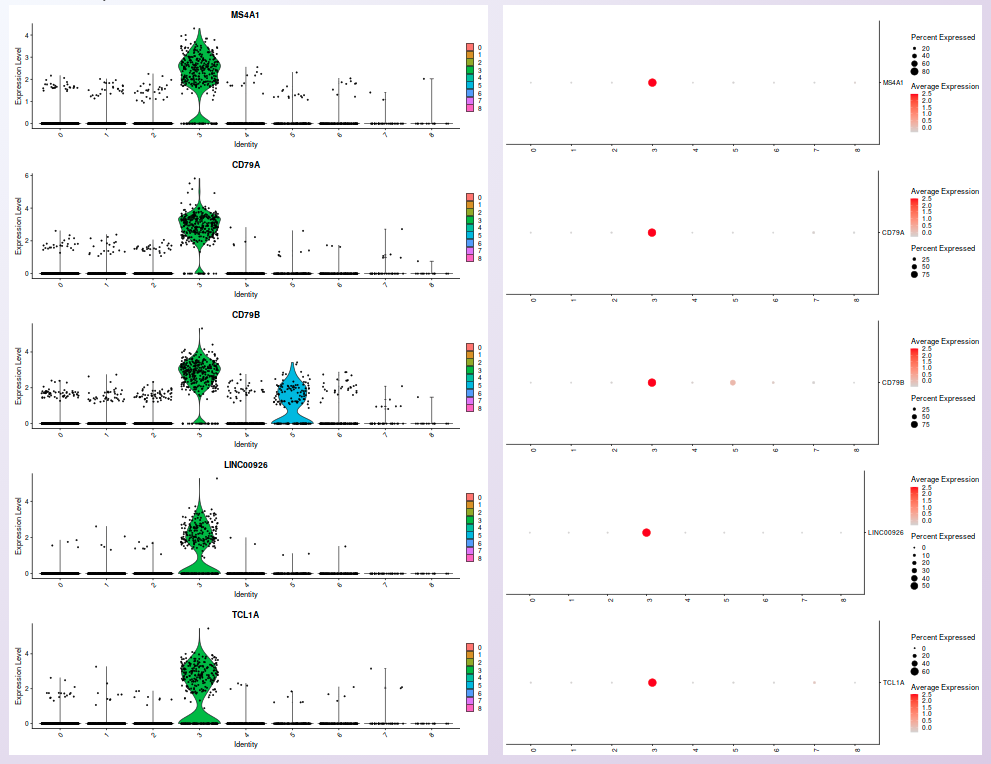


**Figure S7. Asc-Seurat allows an easy way to compare the expression profile of a gene among clusters.** Violin plots show the distribution of cells within each cluster according to the gene expression (left panel), and dot plots show the percentage of cells in each cluster expressing the gene (right panel).


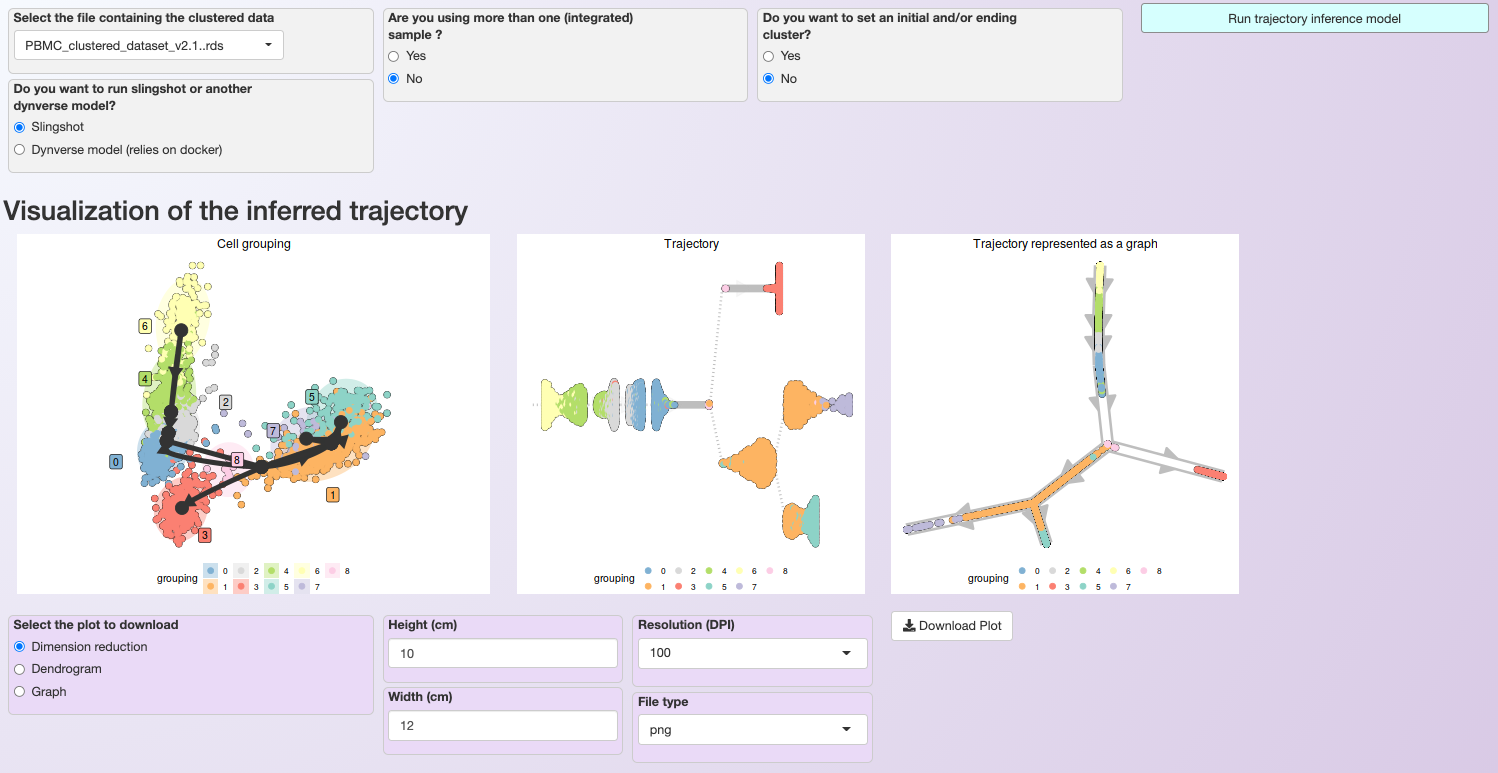


**Figure S8. Asc-Seurat provides multiple models for trajectory inference analysis and three options for trajectory visualization.** Users only need to choose the dataset, the model, and, optionally, to inform the cluster(s) expected to be at the start and/or at the end of the trajectory (top). Three representations of the trajectory are shown, facilitating the interpretation of results (bottom). Moreover, if the dataset corresponds to the integration of multiple samples, it is possible to change the color scheme, so cells are colored by sample instead of by cluster.


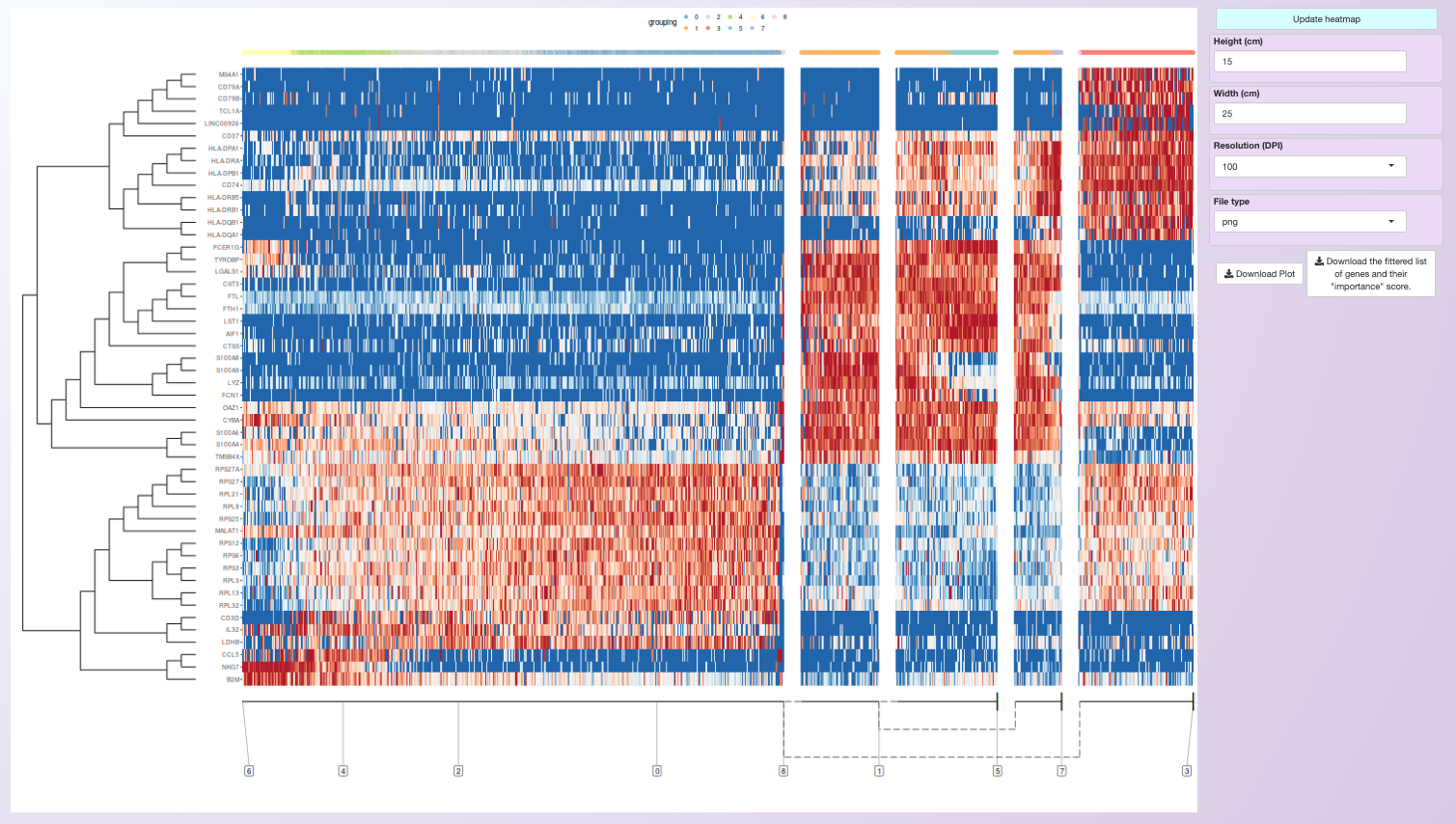


**Figure S9. A heatmap shows the expression of genes within cells by the order these cells appear in the trajectory.** At the bottom of the heatmap, a representation of branches of the trajectory facilitates the interpretation of gene expression profiles. At the top of the heatmap, clusters are represented by the same color scheme as the trajectory representation described previously.


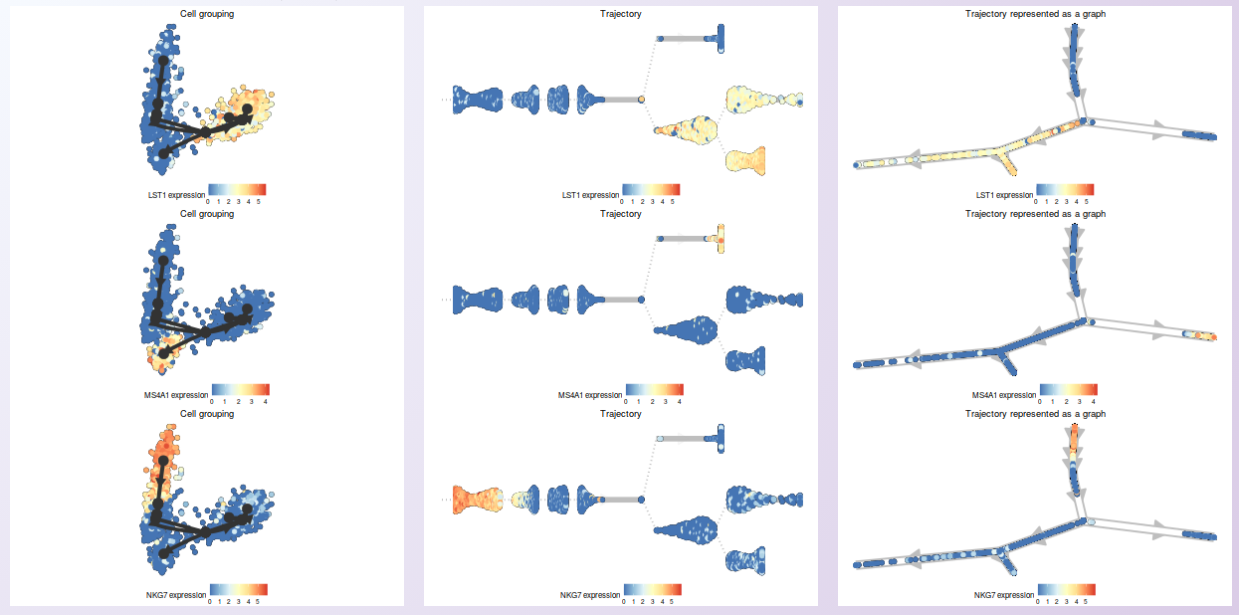


**Figure S10. Asc-Seurat allows users to select genes to visualize their expression profile at the cell level and within the trajectory.** For each gene, three trajectory representations are generated, showing the expression of the genes in the cells ordered in the trajectory.


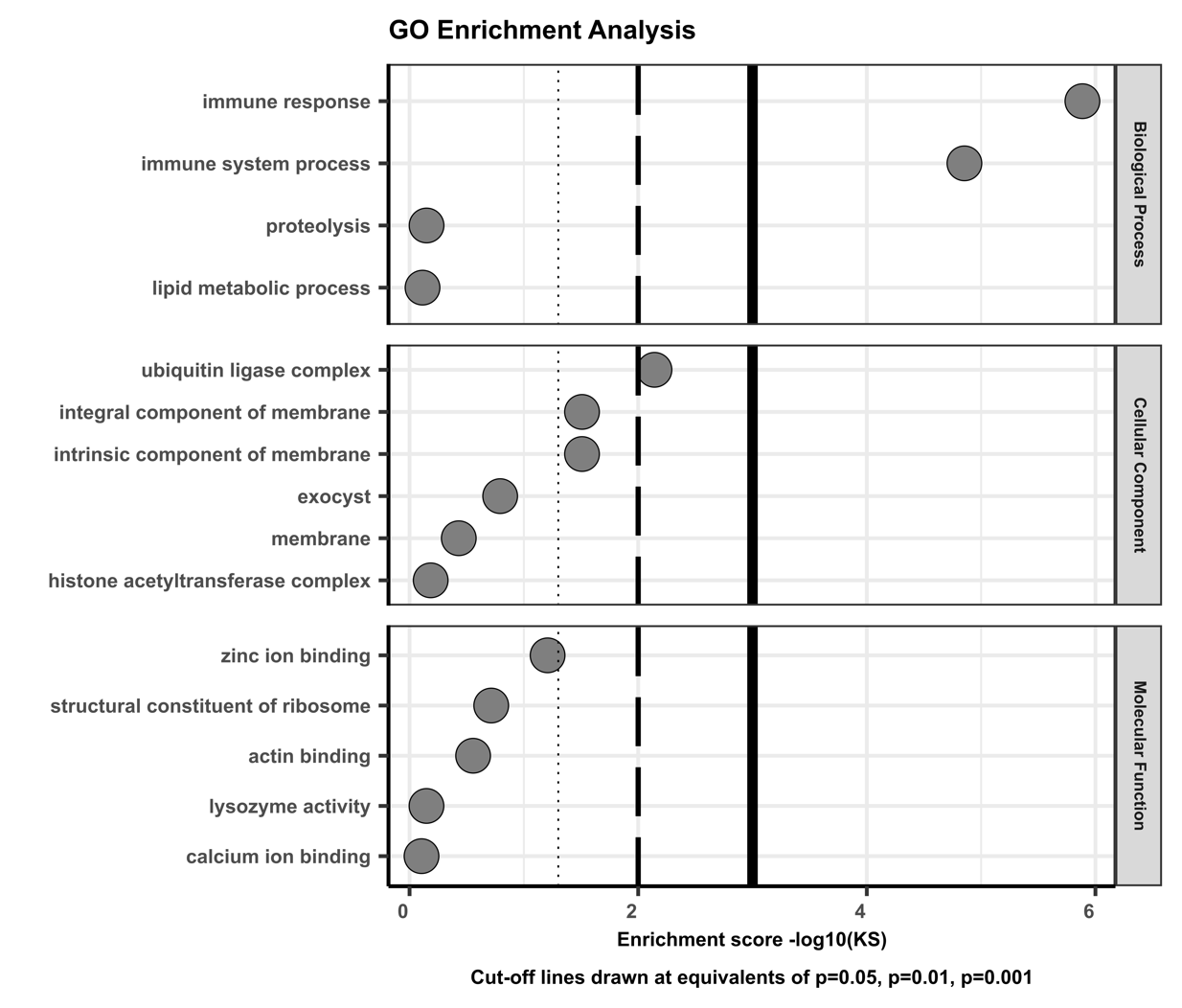


**Figure S11. GO terms enrichment analysis for the set of 50 most important DEGs within the trajectory inferred for the PBMC dataset.**


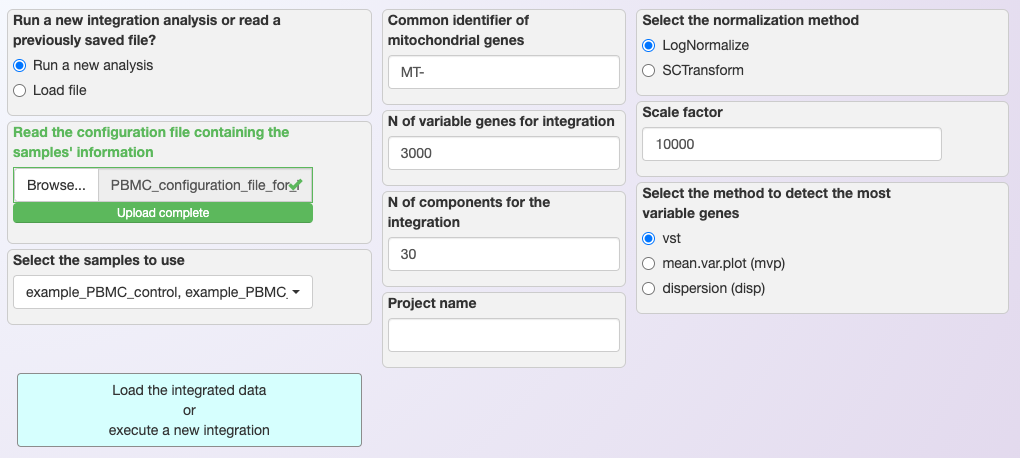


**Figure S12. GO terms enrichment analysis for the set of 50 most important DEGs within the trajectory inferred for the PBMC dataset.**


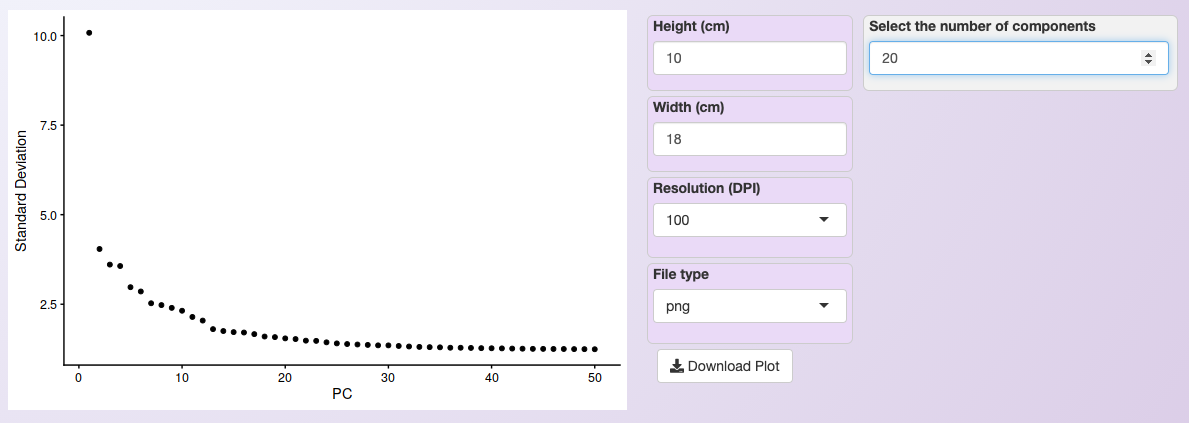


**Figure S13.** **The elbow plot (left) is used to guide the selection of the number of PCs used during the clustering of the PBMC IFN-β dataset (right).**


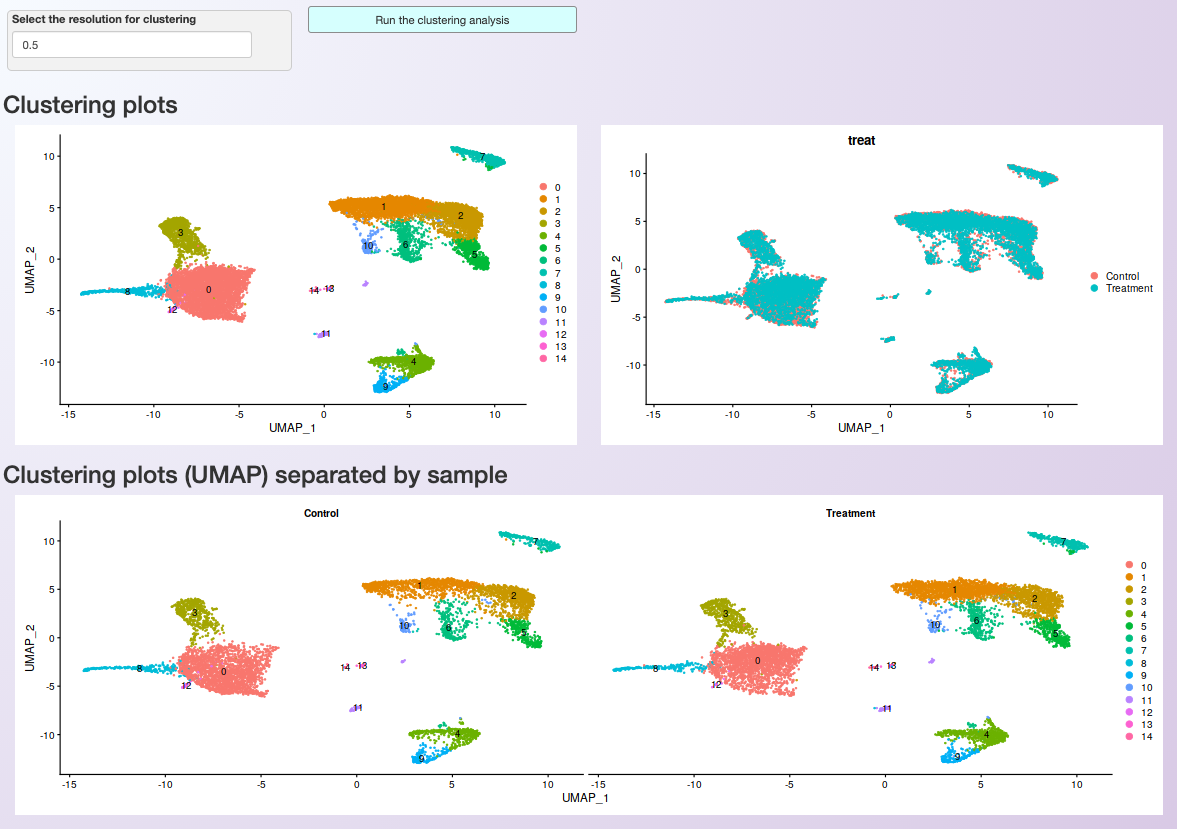


**Figure S14. Cluster visualization of integrated multiple samples.** For the PBMC IFN-β dataset, a resolution of 0.5 was defined (top). The plot is generated using the Uniform Manifold Approximation and Projection (UMAP) technique and divided to show the cells population of each sample (bottom left). Fifteen clusters were obtained for the PBMC IFN-β dataset.


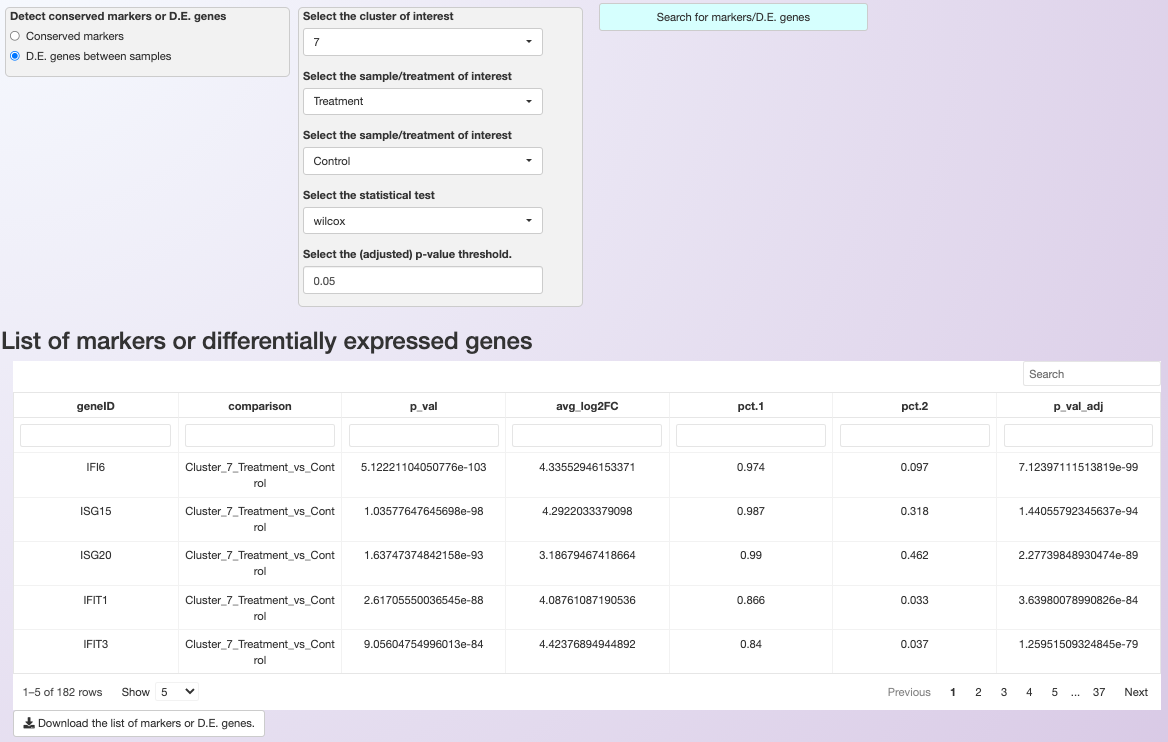
**Figure S15. Identification of DEGs among samples in the integrated dataset.** When using an integrated dataset, user can search for gene markers in each cluster and conserved among samples. Also, it is possible to identify DEGs among samples for each cluster. The image shows the top five PBMC IFN-β DEGs in the comparison between treatment and control for cluster 7.


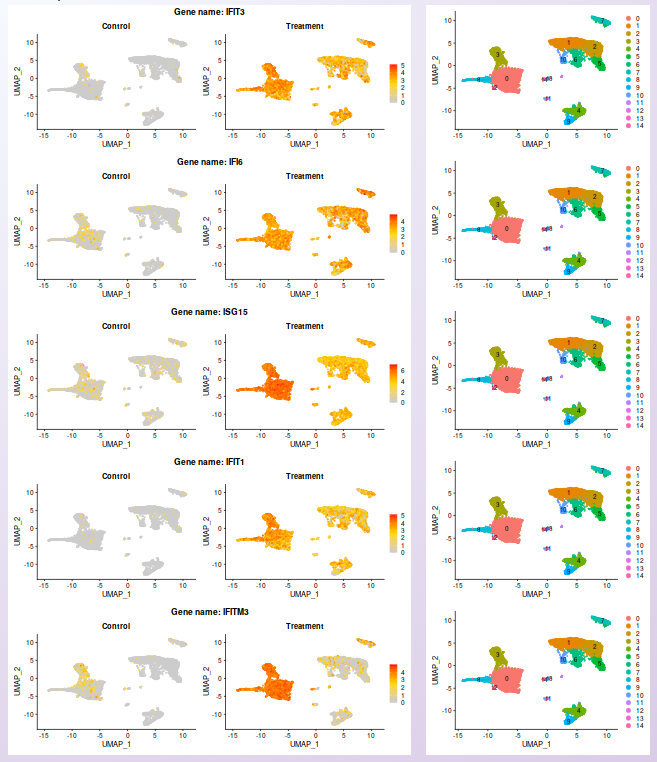


**Figure S16. Visualization of the gene expression at the cell level when using multiple samples.** The image shows the top five PBMC IFN-β DEGs in the comparison between treatment and control for cluster 7.


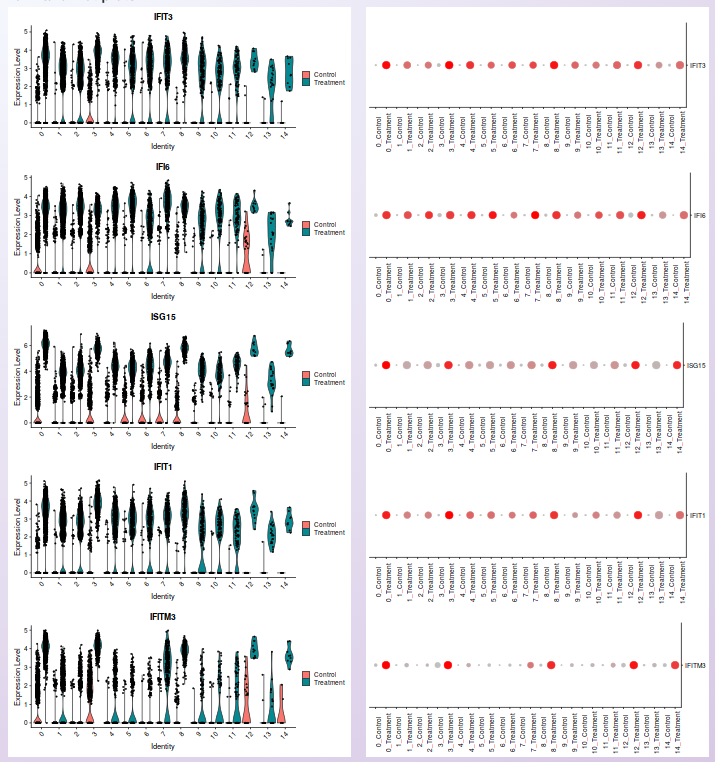
**Figure S17. Violin and dot plots allow the comparison of the expression profile of a gene among clusters and samples.** The image shows the top five PBMC IFN-β DEGs in the comparison between treatment and control for cluster 7.

**
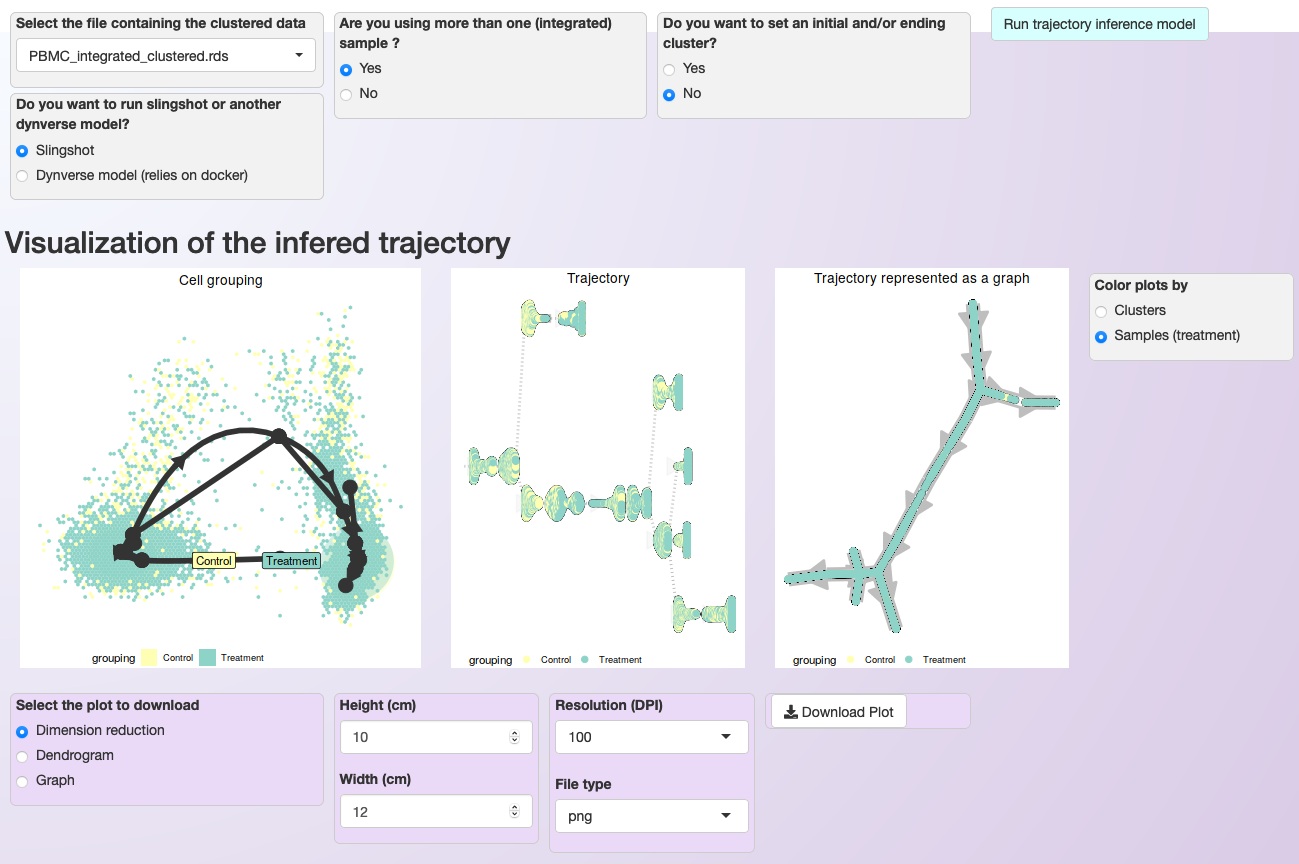
**

**Figure S18. Integrated trajectory colored by sample.**
